# Red and blue light-specific metabolic changes in soybean seedlings

**DOI:** 10.3389/fpls.2023.1128001

**Published:** 2023-03-01

**Authors:** You Jin Lim, Soon-Jae Kwon, Seok Hyun Eom

**Affiliations:** ^1^ Department of Smart Farm Science, College of Life Sciences, Kyung Hee University, Yongin, Republic of Korea; ^2^ Advanced Radiation Technology Institute, Korea Atomic Energy Research Institute, Jeongeup, Republic of Korea

**Keywords:** isoflavone, flavonol, photosynthetic light, metabolic regulation, irradiation period

## Abstract

Red and blue artificial light sources are commonly used as photosynthetic lighting in smart farm facilities, and they can affect the metabolisms of various primary and secondary metabolites. Although the soybean plant contains major flavonoids such as isoflavone and flavonol, using light factors to produce specific flavonoids from this plant remains difficult because the regulation of light-responded flavonoids is poorly understood. In this study, metabolic profiling of soybean seedlings in response to red and blue lights was evaluated, and the isoflavone–flavonol regulatory mechanism under different light irradiation periods was elucidated. Profiling of metabolites, including flavonoids, phenolic acids, amino acids, organic acids, free sugars, alcohol sugars, and sugar acids, revealed that specific flavonol, isoflavone, and phenolic acid showed irradiation time-dependent accumulation. Therefore, the metabolic gene expression level and accumulation of isoflavone and flavonol were further investigated. The light irradiation period regulated kaempferol glycoside, the predominant flavonol in soybeans, with longer light irradiation resulting in higher kaempferol glycoside content, regardless of photosynthetic lights. Notably, blue light stimulated kaempferol-3-*O*-(2,6-dirhamnosyl)-galactoside accumulation more than red light. Meanwhile, isoflavones were controlled differently based on isoflavone types. Malonyl daidzin and malonyl genistin, the predominant isoflavones in soybeans, were significantly increased by short-term red light irradiation (12 and 36 h) with higher expressions of flavonoid biosynthetic genes, which contributed to the increased total isoflavone level. Although most isoflavones increased in response to red and blue lights, daidzein increased in response only to red light. In addition, prolonged red light irradiation downregulated the accumulation of glycitin types, suggesting that isoflavone’s structural specificity results in different accumulation in response to light. Overall, these findings suggest that the application of specific wavelength and irradiation periods of light factors enables the regulation and acquisition of specialized metabolites from soybean seedlings.

## 1 Introduction

Soybean is an economically important crop for oil and protein production and a predominant source of isoflavones in dietary supplements. In East Asian countries such as Korea and Japan, soybean sprouts are commonly used as culinary foods due to their short-term production and high nutritional value. During seed germination, many biochemical changes occur, resulting in the accumulation of various primary and secondary metabolites (Shu et al., 2008; Na Jom et al., 2011; Ma et al., 2022). Flavonoids, particularly isoflavones, which represent the health-promoting properties of soybeans, are synthesized throughout the whole soybean plant (Vacek et al., 2008; Sugiyama et al., 2017; Křížová et al., 2019). Isoflavone is a class of flavonoids that is abundant in soybean seeds and has a protective effect against breast cancer, prostate cancer, cardiovascular disease, and osteoporosis (Anthony et al., 1998; Arjmandi and Smith, 2002; Wei et al., 2012; Křížová et al., 2019). In soybean leaves, kaempferol glycosides are present as major flavonols (Ho et al., 2002; Kim et al., 2008). Kaempferol and its glycosides have health-promoting effects, such as antioxidant, antinociceptive, and anti-inflammatory properties (De Melo et al., 2009; Wang et al., 2018). Despite numerous studies on flavonoids, including isoflavones in soybeans, the regulation of specific flavonoid metabolism is still required for application on artificial controlled agricultural system (Shah and Smith, 2020; Sohn et al., 2021; Wang et al., 2022).

Recent research has focused on the application of artificial light to plants in controlled agricultural systems to produce high levels of phytochemicals (Bian et al., 2015; Zhang et al., 2020). Light is an important abiotic factor that directly or indirectly influences flavonoid biosynthesis in plants (Thoma et al., 2020; Naik et al., 2022). Red and blue artificial light sources are commonly used as photosynthetic lights in smart farm facilities, as they stimulate the production of phytochemicals and the consequent accumulation of various primary and secondary metabolites (Thoma et al., 2020). Red and blue light are the most effectively absorbed light spectra by photosynthetic pigments (Li et al., 2020). While the application of blue light and ultraviolet light to germinating soybeans promotes isoflavone accumulation, it also induces the accumulation of unsaturated fatty acids and free amino acids with a reduced sugar content (Azad et al., 2018; Ma et al., 2018; Ma et al., 2020; Lim et al., 2021). Although short-wavelength light, such as blue light and ultraviolet light, has been effectively used to increase isoflavone levels in soybean plants, red/far-red-mediated phytochrome response has also been reported to contribute to isoflavone level regulation (Kirakosyan et al., 2006).

The relationship between the light environment and flavonoid accumulation has been widely studied (Zoratti et al., 2014; Bian et al., 2015; Fu et al., 2016). Nevertheless, as the use of artificial light increases, studies on metabolic changes and acquisition of target metabolites under light-controlled systems are still required. The relationship between each metabolic pathway in a plant is intricate and strongly influenced by the external environment (Di Ferdinando et al., 2012). Reports on the regulation of flavonoid metabolic steps in response to light are highly dispersed (Neugart et al., 2021; Naik et al., 2022), making it difficult to apply light factors that can control specific metabolites in a certain crop. Moreover, light-induced metabolic changes are highly variable based on crop species and metabolite types (Bian et al., 2015; Thoma et al., 2020). In smart farms and plant factory facilities, the regulation of specialized metabolites, notably secondary metabolites and functional compounds, is essential for the production of high-value-added crops.

A metabolomic approach, including metabolite profiling and specific metabolic gene regulation, is considered a promising strategy for understanding the metabolic regulation by photosynthetic light (Kusano et al., 2011; Li et al., 2019). Although the soybean plant contains both isoflavone and flavonol as major flavonoids (Lim et al., 2021), using light factors to produce a specific flavonoid from this plant remains challenging because the regulation of light-responded isoflavone and flavonol remains unclear. Therefore, the aims of the present study are to evaluate the changes in metabolites in soybean seedlings in response to red and blue photosynthetic lights and to clarify the regulation of isoflavone and flavonol accumulation by different light irradiation periods.

## 2 Materials and methods

### 2.1 Cultivation of soybean seedlings under light treatment

The soybean seeds (*Glycine max* cv. Pungwon) were provided by Pulmuone Food Co. (Chungbuk, South Korea). The soybean seeds were soaked for 4 h with distilled water. The soaked seeds were grown in a 103.0 (diameter) x 78.6 mm (height) plant culture dish (SPL, Pocheon, Korea) with holes of 5 mm in diameter in the bottom under hydroponic culture supplying daily exchanged distilled water. The plant culture dishes were placed in a growth chamber with 23 ± 2°C. The soaked seeds were grown for 7 days in the dark growth chamber for the control group. In order not to affect seed germination by light, the light was irradiated for a total of 5 days after radicle emergence of initial 2 days. For evaluating the effect of LED red (650 nm) and blue (447 nm) light irradiation, 2-day-old soybean seedlings in darkness were transferred to either red or blue light and harvested after 5 d cultivation defined to long-term irradiation in this experiment. On the other hand, 5.5 and 6.5-day-old seedlings in darkness were transferred to the lights and harvested after 36 and 12 h cultivation, respectively, defined to short-term irradiation.

The light sources used in this experiment were red (650 nm) and blue (447 nm) LEDs (D9RBN10SC, Plant Husbandry, Suwon, Korea) (**Supplementary Figure 1**). The intensity of the red and blue LEDs was set to 50 μmol m^−2^ s^−1^ using a photo-radio meter (HD 2302.0; Delta OHM SRL, Marconi, Italy). Each treatment was performed in three replications, and 40 seeds were planted per replicate. The sprouts grown for 7 days were immediately put in liquid N_2_ and stored at −80°C after harvest until the analysis of metabolic gene expression. For the analysis of chlorophyll, GC-MS, and HPLC, the harvested samples were lyophilized at −80°C using a freeze dryer (IlshinBioBase Co. Ltd., Dongducheon, Korea).

### 2.2 Measurement of seedling growth and total chlorophyll content

Sprout growth was measured by shoot and root length and dry weight. The dry weight was measured after the drying process using a freeze dryer (Ilshin Lab. Co., Yangju, South Korea). For chlorophyll analysis, the ground cotyledon of dried seedlings (10 mg) was immersed in 1 mL of 80% aqueous acetone (*v/v*). Chlorophyll was extracted in a shaking incubator for 24 h at 30°C. The extract was centrifuged at 12,000 rpm for 5 min. The absorbance of the supernatant was measured at 663 and 645 nm using a spectrophotometer (S-4100; SCINCO Co., Ltd., Seoul, South Korea). The total chlorophyll content was calculated using the following formula: total chlorophyll (mg g^−1^ DW) = ([8.02 × OD_663_] + [20.2 × OD_645_])/10 (where DW and OD indicate dry weight and optical density, respectively) (Mackinney, 1941).

### 2.3 Determination of metabolites by gas chromatography-mass spectrometry

Untargeted metabolites were analyzed by GC-MS. The samples were derivatized before GC-MS analysis. Derivatization and GC-MS processes were performed using previously published methods, with modifications (Gu et al., 2017; Park et al., 2022). Five milligrams of freeze-dried samples were mixed with 200 μL of 20,000 ppm methyl hydroxyl chloride amine in pyridine solution and treated with sonication for 10 min. The mixture was incubated at 30°C for 90 min for an oxygenation procedure. Fifty microliters of the oximated samples were mixed with 50 μL of a mixture of N,O-bis(trimethylsilyl)trifluoroacetamide and 1% trimethylchlorosilane solution for trimethylsilylation. As an internal standard, 10 μL of 500 ppm fluoranthene was added, and the mixture was vortex mixed and heated for 30 min at 60°C.

The separation of chemical compounds in the derivatized samples, as well as their identification, was performed using gas chromatography and analyzed using a mass selective detector (Agilent Technologies, Palo Alto, CA, USA) operating in selected ion monitoring (SIM) mode. GC (7890 B series, Agilent Technologies) was performed by connecting to a 5977 B MS (Agilent Technologies) equipped with a VF-5MS column (60 m length, 0.25 mm i.d., 0.25 µm film thickness, Agilent Technologies). Helium was the carrier gas at a flow rate of 1.5 mL min^−1^. A split injection mode was executed at a ratio of 20:1 at 300°C. The GC oven temperature was held at 50°C for 2 min, raised to 180°C at a rate of 5°C min^−1^, and then held at the temperature for 8 min. After that, the temperature was increased to 210°C at a rate of 2.5°C min^−1^ and to 320°C at a rate of 5°C min^−1^. The final temperature was held for 10 min. Mass spectra were obtained at 70 eV through electron ionization. Data were acquired in scan mode. The peaks in the chromatogram were identified on the basis of their mass spectra selected using the database of NIST 17 library (http://www.nist.gov/srd/nist1.htm). Each compound was quantified using the internal standard ratio. The quality of prediction was assessed through match factor and reverse match factor with above 800 score, and probability (%) of the library hits. In addition, the retention index values supported the predicted identity. Triplicate analysis was performed.

### 2.4 Determination of flavonoids by HPLC

Extraction and HPLC analyses of isoflavones and kaempferol glycosides were performed using previously published methods (Lim et al., 2021). The standards of eight isoflavone—two aglycones (daidzein and genistein; LC Laboratories, Woburn, MA, USA), three β-glycosides (daidzin, glycitin, genistin; LC Laboratories), and three malonyl glycosides (malonyl daidzin, malonyl glycitin, malonyl genistin; GenDEPOT, Katy, Texas, USA)—were used with a serial concentration (2.5, 5, 10, and 20 mg/L) to calculate the standard curve for quantifying isoflavones in HPLC analysis based on an external standard method. Kaempferol glycosides were quantified with robinin (kaempferol-3-*O*-robinoside-7-*O*-rhanmoside), a kaempferol triglycoside with λmax 265.0 and 347.1 nm. The robinin standard isolated from kudzu and purified as described in Eom et al. (2018) was used with a serial concentration (1.25, 2.5, 5, and 10 mg/L) to calculate the standard curve for quantifying kaempferol glycosides in this experiment.

### 2.5 Transcript level analysis of genes involved in flavonoid biosynthesis by quantitative RT-PCR

Genes characterized in this experiment included PHENYLALANINE AMMONIA-LYASE 1 (*GmPAL1*), CINNAMATE-4-HYDROXYLASE (*GmC4H*), 4-COUMARATE : COENZYME A LIGASE (*Gm4CL*), CHALCONE SYNTHASE 1 to 8 (*GmCHS1*–*8*), CHALCONE REDUCTASE (*GmCHR*), CHALCONE ISOMERASE 1A TYPE II AND 1B1 TYPE II (*GmCHI1AII* and *1B1II*), ISOFLAVONE SYNTHASE 1 and 2 (*GmIFS1* and *GmIFS2*), FLAVANONE 3-HYDROXYLASE (*GmF3H*), and ISOFLAVONE REDUCTASE (*GmIFR*). The gene-specific primers used for qRT-PCR were obtained from Primer-BLAST in the National Center for Biotechnology Information databases (https://www.ncbi.nlm.nih.gov/tools/primer-blast/) and are listed in **Supplementary Table 1**. Total RNA was isolated from 7-day-old soybean seedlings using a Qiagen RNA isolation kit according to the manufacturer’s protocol. Real-time PCR analysis was performed using first-strand cDNA as a template with the QuantiTect SyBR Green PCR kit (Clontech Laboratories Inc., Mountain View, CA, USA). The PCR threshold cycle number of each gene was normalized to the expression level of soybean EUKARYOTIC ELONGATION FACTOR 1-ALPHA (*GmELF1a*) as a reference gene. The normalized transcript levels were expressed as relative values of the dark-grown level. The relative expression level of the gene was calculated following the method of the 2^−ΔΔCt^ comparative Ct.

### 2.6 Statistical analysis

Each experimental treatment was performed in three replications, and the instrumental analysis was also triplicated per treatment. Two-way analysis of variance was performed to assess differences among light wavelength and irradiation period factors by Fisher’s least significant difference (LSD) test using SAS software (Enterprise Guide 4.3 version; SAS Institute Inc., Cary, NC, USA). *Post-hoc* test was performed after ANOVA to assess significant differences between the light treatments using Tukey’s studentized range (HSD) test at the level of *p* < 0.05. Metabolite data for the heatmap were normalized and plotted using Tbtools V1.098693. Circular heatmap was expressed as relative content among compounds and light treatments within each category and also expressed as relative content among light treatments within each compound.

## 3 Results

### 3.1 Seedling growth and chlorophyll content

**Figure 1:**
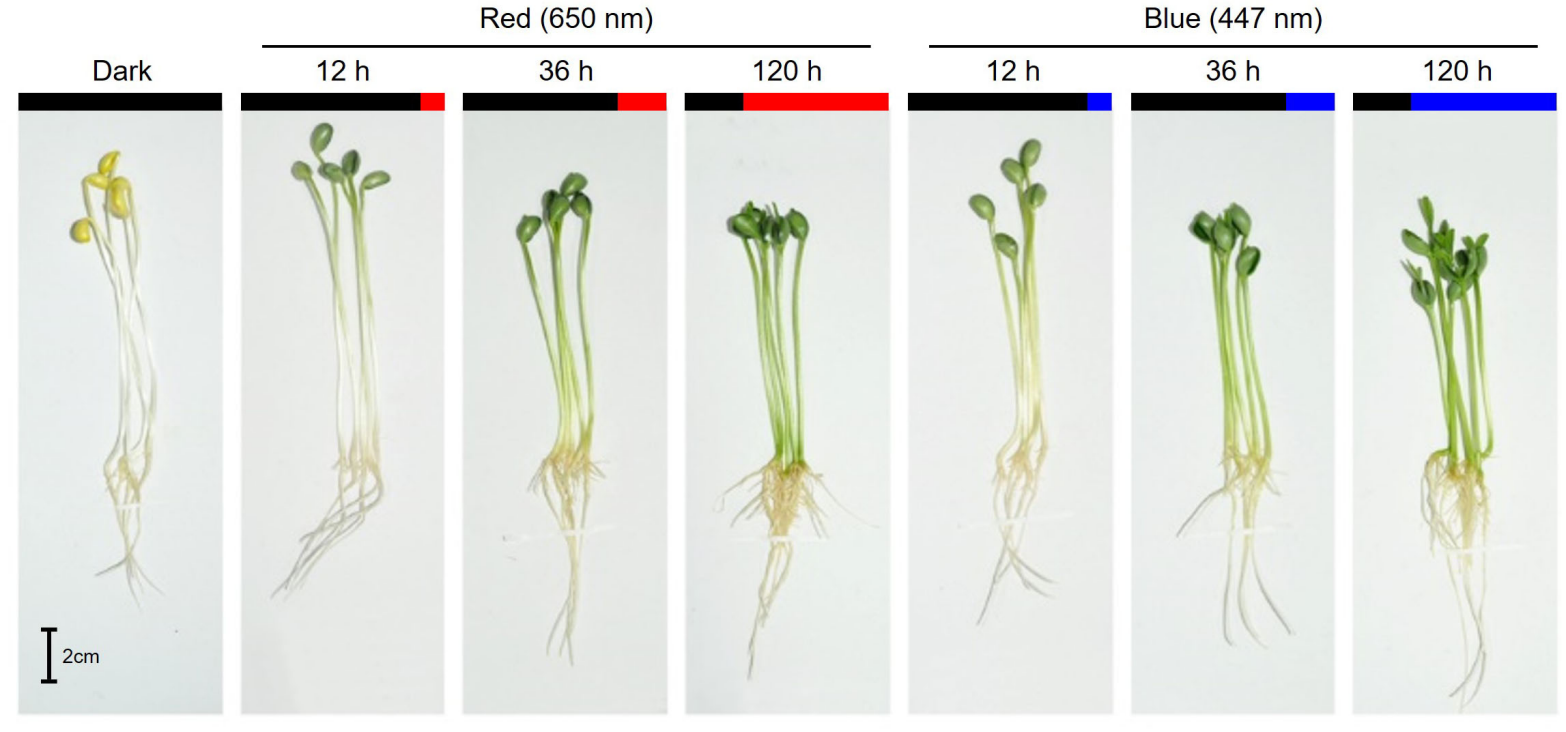
Morphology of 7-day-old soybean seedlings grown under darkness and different exposure times of red (650 nm) and blue (447 nm) light.

Red and blue light strikingly affected shoot elongation (**Figure 1**; **Table 1**). As the irradiation period increased, shoot elongation was reduced, with blue light inhibiting it more than red light during the same irradiation period. While irradiation of both lights for 12 h slightly increased shoot length, long-term irradiation (120 h) of red and blue lights suppressed shoot length by 5% and 34% of dark-grown seedlings, respectively. In contrast, the long-term irradiation of either red or blue lights stimulated the root length compared to dark-grown seedlings. No significant difference was observed in root length between dark and short-term-exposed (12 and 36 h) seedlings.

**Table 1:** Length, dry weight, and total chlorophyll content in soybean seedlings grown under darkness, red (650 nm), and blue (447 nm) light.

Light	Time (h)	Length (cm)		Dry weight (mg)			TCC (mg·g^-1^ DW)
		Shoot	Root	Cotyledon	Hypocotyl	Root	
Dark		12.51 ± 0.26ab	7.20 ± 0.18b	547.1 ± 20.04ab	157.63 ± 5.35b	33.48 ± 0.95c	0.24 ± 0.01e
Red	12	13.40 ± 0.20a	7.22 ± 0.21b	520.01 ± 22.11ab	219.05 ± 6.04ab	50.34 ± 1.32bc	1.14 ± 0.09d
	36	12.31 ± 0.20ab	7.49 ± 0.17b	575.59 ± 11.34a	292.94 ± 75.56a	67.64 ± 3.95ab	3.10 ± 0.10a
	120	11.92 ± 0.89ab	9.43 ± 0.19a	583.23 ± 31.83a	226.93 ± 1.00ab	83.91 ± 3.90a	2.83 ± 0.06a
Blue	12	13.26 ± 0.24a	6.89 ± 0.25b	434.17 ± 2.53b	231.65 ± 8.98ab	64.67 ± 8.73ab	1.10 ± 0.06d
	36	11.46 ± 0.16b	6.65 ± 0.19b	448.78 ± 4.93b	216.41 ± 6.52ab	69.22 ± 4.89ab	1.76 ± 0.01c
	120	8.22 ± 0.33c	9.45 ± 0.21a	498.41 ± 36.06ab	189.34 ± 23.54b	67.39 ± 1.50ab	2.12 ± 0.07b

Different letters (a-e) in each column indicate significant differences at p < 0.05 by Tukey’s studentized range (HSD) test. TCC, total chlorophyll content in cotyledon; DW, dry weight.

The cotyledon dry weight was slightly increased as the red light irradiation period increased but was decreased by blue light compared to dark regardless of period. The hypocotyl dry weight followed a similar pattern as the shoot length. The total chlorophyll content (TCC) in cotyledon increased as the red and blue light irradiation periods increased. The TCC content remained unchanged for both lights for 12 h irradiation, but red light increased TCC relative to blue light at 36 and 120 h irradiation.

### 3.2 Metabolites profiles in soybean seedlings under red and blue lights

**Figure 2:**
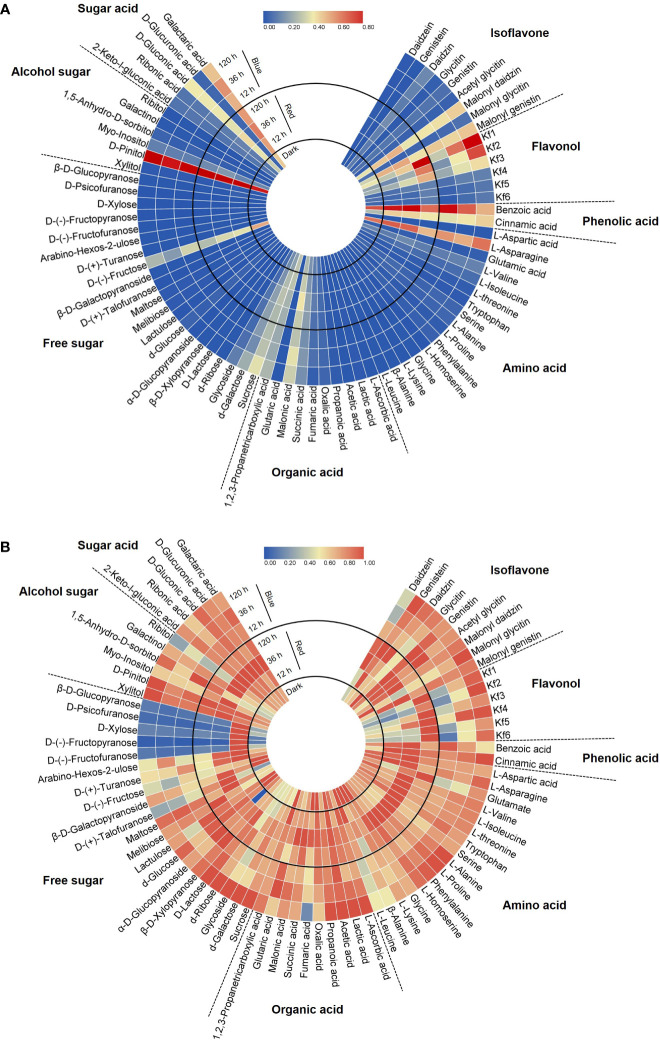
Circular heatmap based on the relative content in each category **(A)** and the relative content in each compound **(B)**. Metabolite data for the heatmap were normalized and plotted using Tbtools V1.098693. Kf1, kaempferol-3-*O*-glycosyl(1→2)-rhamnosyl(1→6)-galactoside; Kf2, kaempferol-3-*O*-(2,6-dirhamnosyl)-galactoside; Kf3, kaempferol-3-*O*-digalactoside; Kf4, kaempferol-3-*O*-diglucoside; Kf5, kaempferol-3-*O*-rhamnosyl-galactoside; Kf6, kaempferol-3-*O*-rutinoside.

A total of 75 metabolites were identified in 8 categories—9 isoflavones, 6 flavonols, 2 phenolic acids, 16 amino acids, 10 organic acids, 21 free sugars, 6 alcohol sugars, and 5 sugar acids—by HPLC and GC-MS analysis. The circular heatmaps clearly show differences in the content among metabolites in each category and among the light treatment groups of each metabolite (**Figure 2**). The predominant metabolites and light-responded metabolites within each category were identified (**Figure 2A**). Notably, asparagine was dominant among the amino acids (61.8%–71.1% of total amino acids) in soybean seedling components. Fructose (25.2%–53.9% of total free sugars) and sucrose (20.3%–39.9%) were dominant among the free sugars. D-pinitol (82.7%–87.5% of total alcohol sugars) and galactaric acid (44.9%–49.2% of total sugar acids) were the dominant alcohol sugar and sugar acid, respectively. These dominant compounds were the most abundant under both light and dark conditions.

**Figure 2B** shows the different accumulation patterns of each metabolite following red and blue light irradiation. Even compounds in the same category showed different light responses depending on the presence or absence of light, light wavelength, and light irradiation period. In general, the light-induced accumulation pattern of metabolites was observed in the following three patterns: increase with short-term irradiation of red (12, 36 h) in this experimental design, increase with long-term irradiation of blue, or decrease following light irradiation. Certain sugar types, such as D-(-)-fructofuranose and D-xylose, were kept in higher accumulation under short-term red light irradiation (**Figure 2B**). Among the eight categories, flavonols were the most significant components that were increased by red and blue light irradiation. Also, it was observed that some specific isoflavones and phenolic acids were light induced metabolites. Therefore, to further reveal the isoflavone–flavonol regulatory mechanism in response to light, the metabolic gene expression level and accumulation of individual isoflavone and flavonol were investigated.

### 3.3 Changes in isoflavone and kaempferol glycoside

**Figure 3:**
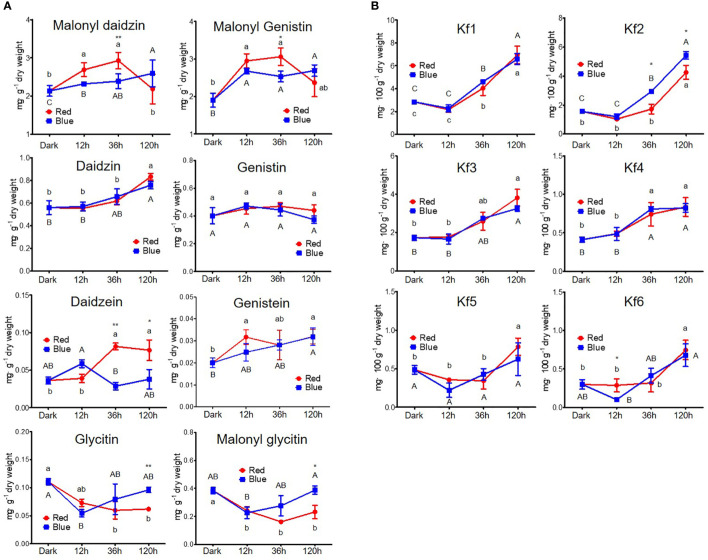
Content of flavonoids, including isoflavone **(A)** and kaempferol glycoside **(B)**. The following abbreviations indicate Kf1, kaempferol-3-*O*-glycosyl(1→2)-rhamnosyl(1→6)-galactoside; Kf2, kaempferol-3-*O*-(2,6-dirhamnosyl)-galactoside; Kf3, kaempferol-3-*O*-digalactoside; Kf4, kaempferol-3-*O*-diglucoside; Kf5, kaempferol-3-*O*-rhamnosyl-galactoside; Kf6, kaempferol-3-*O*-rutinoside; RE, robinin equivalent. Different lower and upper case letters (a–c/A-C) indicate significant differences at *p* < 0.05 by Tukey’s studentized range (HSD) test between irradiation periods within each red and blue light, respectively. Asterisks indicate statistically significant differences (*p < 0.05; **p < 0.01) between red and blue light by Fisher’s LSD test.

**Figure 3** shows the changes in the content of eight isoflavones and six kaempferol glycosides. The contents of malonyl daidzin and malonyl genistin, which were dominant in soybean seedlings, significantly increased by 25.7% and 54.8% in 12 h and by 36.8% and 60.7% in 36 h red light irradiation compared to dark but did not change in long-term irradiation (**Figure 3A**). However, blue light accumulated two malonyl glycosides in different patterns. Malonyl daidzin content increased time-dependently by blue light with a maximum content in 120 h (121.2% of dark-grown seedlings), whereas malonyl genistin content was maintained after increasing at 12 h (140.5% of dark-grown seedlings). The accumulation patterns of seven isoflavones, except for daidzin and genistin, differed between the red and blue lights. Both red and blue light increased daidzin content but did not affect genistin content. Aglycone (daidzein and genistein) showed different patterns between red and blue light, although they were contained in small trace amounts in the seedlings. Red light promoted genistein accumulation more than daidzein in the short term. Conversely, in blue light, daidzein content increased in short-term irradiation, whereas genistein content increased in long-term irradiation. In particular, the content of glycitin-type isoflavones (glycitin and malonyl glycitin) gradually decreased as the red light irradiation period increased, whereas the content decreased for 12 h of blue light irradiation and gradually reversed following prolonged irradiation.

Kaempferol glycosides were controlled by a light irradiation period, exhibiting higher kaempferol glycoside content on longer light irradiation, regardless of photosynthetic lights (**Figure 3B**). Kaempferol glycoside contents peaked at 120 h irradiation, showing 1.62- to 2.98-fold higher in red light and 1.29- to 3.48-fold higher in blue light depending on kaempferol glycoside types than in dark-grown seedling. Kaempferol-3-*O*-glycosyl(1→2)-rhamnosyl(1→6)-galactoside (Kf1) and kaempferol-3-*O*-(2,6-dirhamnosyl)-galactoside (Kf2) were the predominant kaempferol glycosides in soybean seedlings, as previously reported (Lim et al., 2021). They did not respond to short-term irradiation of 12 h but thereafter significantly increased time-dependently. Five kaempferol glycosides were increased time-dependently by both red and blue light, except for kaempferol-3-*O*-diglucoside (Kf4). Kf4 did not increase after 36 h in either red or blue light irradiations. Notably, the accumulation of Kf2 was primarily stimulated by blue light compared to red light, with 1.7- and 1.2-fold higher content in 36 and 120 h irradiation of blue light than red light.

### 3.4 Changes in biosynthetic gene expression and accumulation of flavonoids

**Figure 4:**
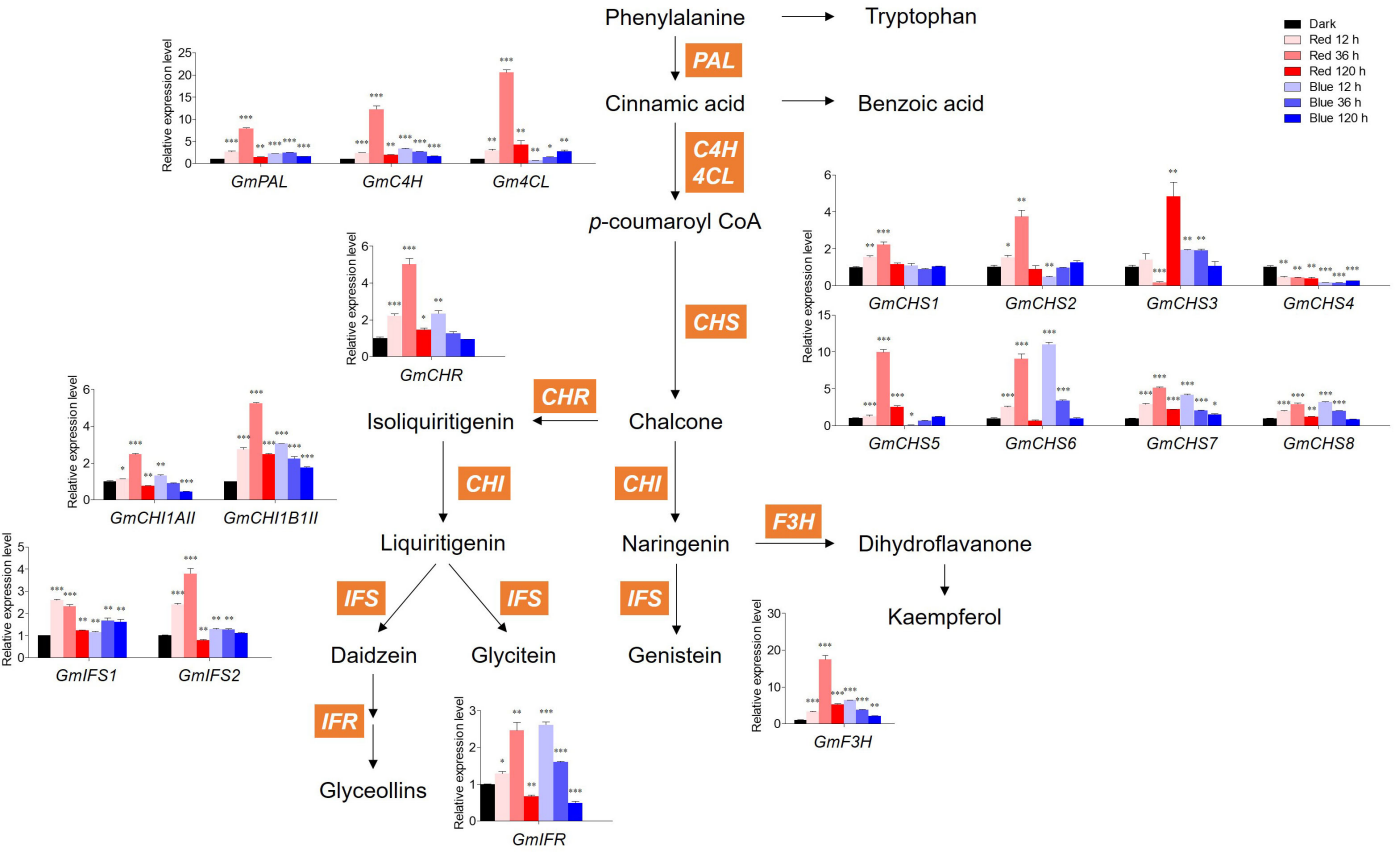
Biosynthetic gene expression associated with flavonoids. The transcript levels were normalized with the reference gene *GmELF1a* and expressed as relative values of the dark-grown level. The following abbreviations of genes indicate *PAL*, phenylalanine ammonia lyase; *C4H*, Cinnamic acid 4-hydroxylase; *4CL*, 4-coumarate:CoA ligase; *CHS*, chalcone synthase; *CHR*, chalcone reductase; *CHI1AII*, chalcone isomerase 1A Type II; *CHI1B1II*, chalcone isomerase 1B1 Type II; *IFS*, isoflavone synthase; *IFR*, isoflavone reductase; *F3H*, flavanone 3-hydroxylase. Asterisks indicate statistically significant differences compared with dark in each gene (*p < 0.05; **p < 0.01; ***p < 0.001) by Fisher’s LSD test.

**Figure 4** shows the expression levels of *GmPAL, GmC4H, Gm4CL, GmCHS1–8, GmCHR, GmCHI1AII* and *1B1II, GmIFS1–2, GmIFR*, and *GmF3H* genes involved in flavonoid biosynthesis. In red light, the expression levels of all genes except *GmCHS3/4* and *GmIFS1* gradually increased up to 36 h and then decreased at 120 h, which was similar to the accumulation patterns of malonyl daidzin and malonyl genistin contents. In blue light, the expression levels of most genes were downregulated time-dependently after upregulation in short-term irradiation (12 h), whereas those of *Gm4CL*, *GmCHS2/5*, and *GmIFS1* were gradually upregulated time-dependently.

Concerning *GmCHSs*, the *GmCHS1–5* expression levels were averagely higher in red light than in blue light. However, the *GmCHS6/7/8* expression levels responded to 12* h* blue light irradiation, showing a high level similar to that of 36 h red light irradiation. Among *CHSs*, *GmCHS5–6* expression levels were the highest in each 36 h of red light and 12* h* of blue light, with 10- and 11-fold higher expression than in dark, respectively. Interestingly, *GmCHS4* expression was inhibited by both red and blue light and had a lower level than that in dark conditions. Genes involved in isoflavone synthesis, *GmIFS1–2*, displayed distinct patterns under red and blue light. Under red light irradiation, the *GmIFS2* expression level was highest at 36 h, whereas that of *GmIFS1* decreased time-dependently. Under blue light irradiation, *GmIFS1* was highly expressed compared to dark conditions, whereas *GmIFS2* remained unaltered by blue light and was upregulated only by red light.

Red and blue light significantly upregulated *GmF3H* relative to other genes. Red light significantly increased *GmF3H* after 36 h irradiation, with 17-fold higher expression compared to the dark. Most flavonoid biosynthetic genes were significantly more upregulated by short-term red light irradiation than by blue light. However, long-term irradiation of both lights led to the inhibition of flavonoid biosynthetic gene expression, resulting in a temporal discrepancy between gene expression and flavonoid accumulation.

### 3.5 Amino acids, phenolic acids, and free sugars involved in flavonoid synthesis

**Figure 5:**
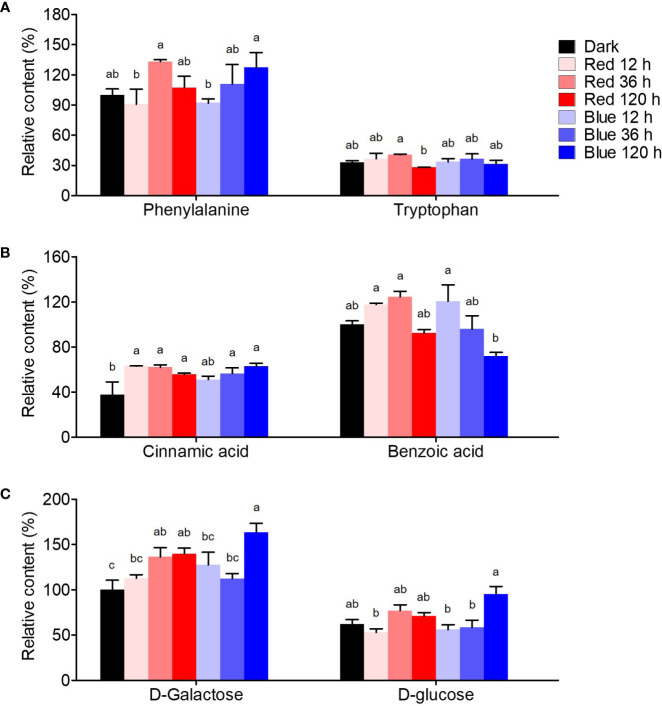
Flavonoid synthesis-related metabolites, including amino acid **(A)**, phenolic acid **(B)**, and free sugar **(C)**. The content of amino acid, phenolic acid, and free sugar was expressed as relative content (%) of phenylalanine, benzoic acid, and D-galactose of dark-grown seedlings in each metabolite group, respectively. Different letters (a–c) above the bars in each metabolite indicate significant differences at *p* < 0.05 by Tukey’s studentized range (HSD) test.

**Figure 5** shows the quantification of flavonoid-related intermediate metabolites. Phenylalanine, a precursor for flavonoid synthesis, showed the highest content at 36 h in red light with a 1.3-fold higher content than in dark-grown seedlings, whereas it maximally increased at 120 h in blue light, presenting similar max content in red and blue lights. Tryptophan, competitively synthesized with phenylalanine, was not significantly affected by either light.

Benzoic and cinnamic acids were accumulated differently by red and blue light in soybean seedlings. Cinnamic acid, a precursor phenolic acid of flavonoids, increased by photosynthetic lights compared to dark conditions, regardless of irradiation period and light wavelength. Cinnamic acid was time-dependently increased by blue light, whereas it rapidly increased in red light short-term irradiation. The contents were 1.7-fold higher than in dark-grown seedlings in both 120 h of blue and 12 h of red light. Meanwhile, benzoic acid, in a competitive pathway with flavonoid synthesis, was considerably affected by the irradiation period. While 12 and 36 h red light irradiation stimulated benzoic acid accumulation, 120 h irradiation did not. However, in blue light, benzoic acid content increased by 20.6% at 12 h compared to dark conditions but decreased time-dependently after that, resulting in a 28% decrease in the content at 120 h.

D-galactose and D-glucose, which are sugar donors for flavonoid glycoside synthesis, accumulated differently under red and blue light. The concentrations of both sugars remained unchanged after 36 h of exposure to red light, while they increased significantly after 120 h of exposure to blue light. The upregulation of free sugar accumulation by photosynthetic light may have contributed to the increased flavonol glycoside content.

## 4 Discussion

### 4.1 Metabolic profiles in response to photosynthetic light and light irradiation periods

Circular heatmap data clearly showed the relative level of photosynthetic light response for a set of metabolites, including amino acids, organic acids, flavonoids, phenolic acids, free sugars, alcohol sugars, and sugar acids. These metabolic profiling results enabled the distinction between light-responsive and nonresponsive metabolites and revealed that the photosynthetic light response of specialized metabolites is linked to several metabolites. Photosynthetic light, such as red and blue light, markedly affects plant metabolism. Light promotes the metabolism of phenylalanine, a key amino acid precursor for flavonoid synthesis, *via* the upregulation of the phenylalanine ammonia lyase reaction (Seo et al., 2015; Liu et al., 2018). Light also increases flavonoid concentrations by activating the biosynthesis and metabolism of lipids (Maldini et al., 2015).

Metabolic profiling provides insight into the degree to which germinating soybean seedlings respond to short-term or long-term irradiation of two different light wavelengths with respect to their chemical composition, including primary and specific secondary metabolites. Even for compounds belonging to the same category, metabolic changes based on the presence or absence of light, light wavelength, and irradiation period showed significant light response differences. Through untargeted metabolomics based on GC-MS and HPLC analysis, specific metabolites that can be controlled by the wavelength and period of light were identified, allowing for further studies on their light response mechanisms.

### 4.2 Light-induced synthesis of two flavonoid branches of isoflavone and flavonol

The specific responses of flavonoids to photosynthetic light have been distinguished from those of other metabolites. Most plants have a common major flavonoid pathway, but they frequently derive specific branches to adapt to variable environmental conditions (García-Calderón et al., 2020). Connecting primary and secondary metabolism, the chalcone synthesis pathway provides precursors for the synthesis of multibranched downstream metabolites (Austin and Noel, 2003). Two branches of flavonoids, isoflavone and flavonol, are derived from chalcone. Although many studies have explored the effect of light on isoflavone production in soybeans, the light-controlled coregulation of the isoflavone and flavonol pathways remains underexplored. Our results revealed that the synthesis of kaempferol glycosides was highly dependent on red and blue irradiation periods, whereas isoflavone accumulation was type-dependent in response to each light quality.

Light-induced isoflavone accumulation was regulated differently based on the isoflavone type. Type-dependent synthetic pathways exist for isoflavone. The genistein synthetic pathway differs from that of daidzein and glycitein (**Figure 4**), where genistein is from naringenin while daidzein and glycitein are from isoliquiritigenin (Ralston et al., 2005). In addition, different types of isoflavone are organ-specifically accumulated, presenting different isoflavone compositions in each organ of soybean plants (Graham, 1991; Yang et al., 2020; Lim et al., 2021). Although genistin and malonyl genistin were increased by photosynthetic light, they did not differ significantly from other isoflavone types. Regarding the chemical structure of isoflavones, glycitein has a methoxy group, unlike daidzein and genistein. According to Fu et al. (2016), flavonol methyl derivatives in tobacco negatively correlate with ultraviolet light and red/far-red light. Red and blue light irradiation decreased the glycitein type in the present study, validating the negative correlations between isoflavone methyl derivatives and photosynthetic light. Long-term red light irradiation induced a continuous decrease in the glycitein type, indicating that the negative correlation is more dependent on red light. In addition, red and blue light-responded accumulation of isoflavone in soybean seedlings was limited to daidzein and genistein types, corroborating previous reports (Azad et al., 2018). These differences in synthetic pathways, tissue-specific accumulation, and chemical structures would have resulted in different light-responded accumulations depending on each isoflavone type.

Several studies have shown the enhancing effects of blue light and ultraviolet light on various flavonoids, including isoflavone (Jenkins et al., 1995; Zoratti et al., 2014; Taulavuori et al., 2018). However, little is known about the effect of red light on isoflavones. Specific red-light-responsive isoflavones remain unexplored, but red light has been shown to increase isoflavone glycoside content without affecting isoflavone aglycones (Kirakosyan et al., 2006). In addition, red and far-red light influenced isoflavone accumulation based on the shade tolerance level of soybean plants, with a decrease in the red/far-red ratio increasing leaf isoflavones and reducing hypocotyl isoflavones in high shade-tolerance soybeans (Qin et al., 2017). Although the isoflavone accumulation pattern in response to light quality varied depending on the type, this study found that short-term red light irradiation could be efficient for producing malonyl daidzin and malonyl genistin, the dominant isoflavone in soybeans. Long-term red light irradiation, however, negatively affected the production of glycitein-type isoflavones.

All kaempferol glycoside types increased in response to photosynthetic light. The accumulation of kaempferol glycosides was highly dependent on the light irradiation period, indicating that the accumulation of flavonol is more light-dependently regulated than isoflavone. Although the content of all kaempferol species was increased by red and blue light, Kf2 production was promoted by blue light rather than red light. The regulation of specific kaempferol derivatives by controlling light factors is rarely studied, but blue light promoted the accumulation of kaempferol aglycone compared to red light in Chinese cabbage seedling and lettuce leaf (Kim et al., 2015; Lee et al., 2019). Notably, the attenuation of blue light can result in an increase in certain kaempferol derivatives and a decrease in quercetin derivatives in pea leaves (Sipola et al., 2015). In addition, blue light stabilized the UV-induced high concentration of kaempferol derivatives in kale seedlings, whereas this effect did not apply to kohlrabi seedlings (Neugart et al., 2021). Although both light wavelength and irradiation period have a significant effect on flavonol regulation depending on the crop, our results indicate that light irradiation period rather than the light wavelength is a critical factor for the regulation of kaempferol glycoside accumulation in soybean seedlings.

Kaempferol mainly accumulates in aerial parts that receive direct light in many plants such as soybean, turnip, and fennel, and is not found or in trace amounts in root tissue with or without light (Soliman et al., 2002; Fernandes et al., 2007; Karimi et al., 2011; Lim et al., 2021). Our previous study revealed that ultraviolet light-induced Kf2 accumulation in soybean seedlings occurred primarily in the hypocotyl (Lim et al., 2021). Therefore, blue light-induced Kf2 is inferred by an increase in its content in hypocotyl. Various flavonoids, as well as isoflavones and kaempferol derivatives, are generally regulated by photosynthetic light. It has known that higher light intensity and longer period in red and blue light irradiation induced higher level of anthocyanin in red kale sprouts (Carvalho and Folta, 2014). In addition, flavonoids in buckwheat sprouts, including orientin, isoorientin, vitexin, isovitexin, quercetin 3-*O*-robinobioside, and rutin, were also enhanced by sequential red and blue light irradiation (Nam et al., 2018).

### 4.3 Flavonoid biosynthetic genes in response to red and blue lights

Overall, with some exceptions, flavonoid biosynthetic genes were maximally expressed at 36 h under red light, peaking at 12 h and gradually decreasing under blue light. The expression of many flavonoid biosynthetic genes in soybean seedlings peaked at 36 h of red light, showing a similar pattern to malonyl-type isoflavone accumulation. The expression patterns of *GmCHS2/5* decreased at 12 h under blue light and then gradually increased, consistent with the accumulation pattern of glycitein-type isoflavones, indicating that *GmCHS2/5* are mainly involved in the synthesis of glycitein types only in response to blue light. These results indicate that the time lag between gene expression and metabolite accumulation is dependent on light wavelength. Different gene-to-metabolite patterns depending on light wavelength have also been observed in strawberry fruits (Zhang et al., 2018). Under blue light, strawberry fruits had lower gene expression levels, inconsistent with their high anthocyanin content, whereas under red light, most genes remained active even though the anthocyanin content was low.

The expression of *GmCHSs* in soybean seedlings was highly variable based on the wavelength and period of light. *GmCHSs* were upregulated in response to red light rather than blue light, but exceptionally, *GmCHS6/7/8* were upregulated in response to both red and blue lights. Differences in flavonoid gene expression in response to red and blue lights are explained by differences in photoreceptors and their signaling pathways. Gene expression is regulated at the transcriptional and posttranscriptional levels by light signals detected by photoreceptors and transduced in photosystems (Martınez-Hernández et al., 2002; Floris et al., 2013). Blue light induces *CHS*, which is predominantly mediated by cry 1 and does not require phytochrome. However, red light upregulates cryptochrome 1-mediated *CHS* expression because phytochrome regulates the cry 1 inductive pathway (Wade et al., 2001). Our qRT-PCR results demonstrated that the mechanism by which the *GmCHS* expression level was differentially regulated depended on light wavelength because red and blue lights have distinct signaling pathways. Previous reports have shown that *CHS6/7/8* sensitively responds to ultraviolet light (Lim et al., 2020). UV-A/blue light-induced *CHS* expression in a signal transduction pathway distinct from the UV-B response (Christie and Jenkins, 1996). However, our previous and current studies demonstrated that *GmCHS6/7/8* were significantly upregulated by UV-A/UV-B/blue light compared to other *CHSs* and were highly sensitive to shorter wavelengths, including UV-A/B/blue light.

UV radiation, as a stress factor, can downregulate the expression of flavonoid biosynthesis genes (Lim et al., 2020; Lim et al., 2021). However, our results show that excessive light exposure, regardless of light wavelength, can inhibit the expression level of flavonoid biosynthetic genes. Light intensity and light wavelength strongly influence the expression of genes involved in flavonoid synthesis (Xu et al., 2014). Flavonoid accumulation patterns were not consistent with most gene expression patterns. Long-term light irradiation inhibited flavonoid biosynthetic gene expression, resulting in a periodic discrepancy between gene expression and flavonoid accumulation. The gene-to-metabolite discrepancy is commonly observed and is due to the time lag from gene transcription to metabolite synthesis (Nakabayashi et al., 2017). Nevertheless, the expressions of most genes involved in flavonoid synthesis were highly upregulated by short-term red light irradiation (12 and 36 h) compared to blue light, exhibiting a strong correlation between red light and the accumulation of malonyl types.

### 4.4 The relationship between flavonoids, upstream metabolites, and primary metabolites

Various intermediate metabolites are required and are involved in flavonoid synthesis. The red/blue light-induced increase in phenylalanine, a precursor amino acid of flavonoids, resulted in an increase in flavonol content. Tryptophan, which is in a competitive pathway with phenylalanine, did not change significantly in response to light, indicating that phenylalanine-related metabolism is more light-dependent. Although phenylalanine metabolism contributes to the formation of various metabolites such as proteins, tyrosine derivatives, polyamines, tannins, and lignin as well as flavonoids (Bassard et al., 2010; Barros and Dixon, 2020), it was highly related to flavonoid synthesis in soybean seedlings under red and blue lights. Under blue light, cinnamic acid increased, whereas benzoic acid significantly decreased, indicating that blue light inhibits the synthesis of cinnamic to benzoic acid. The reduction of phenylalanine and cinnamic acid by long-term red light irradiation affected malonyl isoflavone accumulation. Although sugars are involved in various metabolisms, the sufficient provision of free sugars induced by red and blue lights has a significant effect on the increase in flavonoid content. Consequently, the sufficient production of flavonoid precursors induced by red and blue lights and the inhibition of the competitive pathway by blue light resulted in the upregulation of isoflavone and flavonol synthesis in soybean seedlings.

## 5 Conclusion

Here, we suggest that a sequential light irradiation system with single red or blue light is an effective method to regulate the metabolism of specific isoflavone and flavonol in soybean seedling. Among the eight metabolite categories, flavonols were the most significant components that were increased by red and blue light irradiation. Also, it was observed that some specific isoflavones and phenolic acids were light induced metabolites. The light irradiation period rather than the light wavelength was an important factor in the regulation of kaempferol glycoside accumulation. In particular, kaempferol-3-*O*-(2, 6-dirhamnosyl)-galactoside showed higher accumulation under blue light than red light. Higher accumulation of kaempferol derivatives was induced by a longer light irradiation period; however, kaempferol-3-*O*-diglucoside production unexpectedly stopped increasing after 36 hours of both red and blue light. On the contrary, the pattern of the light-responded accumulation of isoflavones considerably varied depending on the type of isoflavone. Red light was effective in increasing total isoflavone levels by inducing high accumulation of malonyl daidzin and malonyl genistin, the main isoflavones of soybean seedling. However, longer irradiation of red light (120 h) caused a reduction of those main isoflavones, indicating that isoflavone is significantly affected not only by the light wavelength but also by the irradiation period. Daidzein increased only in response to red light, while glycitin types decreased in response to the prolonged red light irradiation, suggesting that isoflavone structural specificity results in the accumulation of different isoflavone profiles in response to light. In conclusion, our findings imply that a single red and blue light can be used to selectively regulate isoflavone and flavonol in soybean seedlings and that the light wavelength and irradiation period are critical in deciding which flavonoids are being targeted.

## Data availability statement

The datasets presented in this study can be found in online repositories. The names of the repository/repositories and accession number(s) can be found in the article/**Supplementary Material**.

## Author contributions

SE designed and supervised the project. YL designed the experiment and drafted the manuscript. YL and S-JK performed laboratory experiments and analyzed data. SE participated in the material preparation. SE and S-JK revised the manuscript. All authors contributed to the article and approved the final version of the manuscript.

## Supplementary material

Supplementary Table 1 Gene specific primers sequences for qRT-PCR.

Supplementary Figure 1 Light spectra of the red (A) and blue (B) LEDs used in this experiment.

Supplementary Figure 2 Total ion chromatograms by GC-MS of soybean seedling grown under different light treatments.
