# Supplementary material for: Red and blue light-specific metabolic changes in soybean seedlings
Source: Front Plant Sci. 2023 Mar 1;14:1128001. doi: 10.3389/fpls.2023.1128001 (PMC10014548; doi:10.3389/fpls.2023.1128001)
Supplement: Supplementary Table 1 — Gene specific primers sequences for qRT-PCR. [file DataSheet_1.docx]

Supplementary Material

**Supplementary Table 1.** Gene specific primers sequences for qRT-PCR.

| Gene | Accession number | Description | Forward Primer | Reverse Primer |
| --- | --- | --- | --- | --- |
| *GmPAL* | NM_001357057 | Phenylalanine ammonia-lyase 1 | AGCAACACAACCAGGATGTCAA | CAATTGCTTGGCAAAGTGCA |
| *GmC4H* | NM_001250388 | Cinnamate-4-hydroxylase | AGGCGAGATCAACGAAGACAAC | GTTCACAAGCTCAGCAATGCC |
| *Gm4CL* | NM_001249307 | 4-Coumarate:coenzyme A ligase | AGGCAATGTACGTGGACAAGCT | TCCGAGAGGACAGAGAAGTGGA |
| *GmCHS1* | NM_001350109 | Chalcone synthase 1 | AAGCGCATGTGTGATAAGTCGA | TTGCATCCAACGAAGGTGC |
| *GmCHS2* | NM_001360424 | Chalcone synthase 2 | TATGGCACCTTCATTGGATGC | GCTGGTGGTGCAAAAAATGAG |
| *GmCHS3* | NM_001289367 | Chalcone synthase 3 | GAGATCCGTAATGCACAACGTG | CTTTGAGCTCGGTCATGTGCT |
| *GmCHS4* | XM_014778878 | Chalcone synthase 4 | CCTTCCAAGCCACTTTGCA | CTGGAGCAAAGGATGAAAGTGA |
| *GmCHS5* | NM_001360359 | Chalcone synthase 5 | CACTTTGCCACATTCATTCC | TGTGAATGAACTAATGAAGCTATAGC |
| *GmCHS6* | XM_003533759 | Chalcone synthase 6 | ACCAACAGTGACCACATGAACG | GGCACAAACACTTGGATTCTCC |
| *GmCHS7* | NM_001353380 | Chalcone synthase 7 | AACCCACCAAACCGTGTTGAT | CTTGTCACACATGCGCTGAAAT |
| *GmCHS8* | NM_001317656 | Chalcone synthase 8 | ATGGAGCTGCTGCTGTCATTG | CCTCACGAAGGTGTCCATCAA |
| *GmCHR* | NM_001249044 | Chalcone reductase | CAAAGCCATTGGAGTCAGCAA | CCATGCAAGGTTCATCTCCACT |
| *GmCHI1AII* | NM_001248290 | Chalcone isomerase 1A Type II | GGCGCTGAATACTCAAAGAAGG | AGAGGCACCAGGTGCAAAATT |
| *GmCHI1B1II* | NM_001249826 | Chalcone isomerase 1B1 Type II | AGCTGAATTGCTCGACTCCCT | CAGATTGCATATGTGCCACACA |
| *GmIFS1* | NM_001249093 | Isoflavone synthase 1 | AGAATTCCGTCCCGAGAGGTT | TGCCATTCCTGAAGTAGCCAA |
| *GmIFS2* | NM_001251586 | Isoflavone synthase 2 | AATGTGCCCTGGAGTCAATCTG | GGCGTCACCACCCTTCAATAT |
| *GmIFR* | NM_001254100 | Isoflavone reductase | GAGGAGGATAGTGAGGGCAA | CCTGATGTGAACACCAGAGATG |
| *GmF3H* | A Y669324 | Flavanone 3-hydroxylase | CCGTTTGTCCATAGCCACTT | GCCTCAAGTTTTGCCTTCTG |
| *GmELF1a* | NM_001250496 | Eukaryotic elongation factor 1-alpha | GACCTTCTTCGTTTCTCGCA | CGAACCTCTCAATCACACGC |


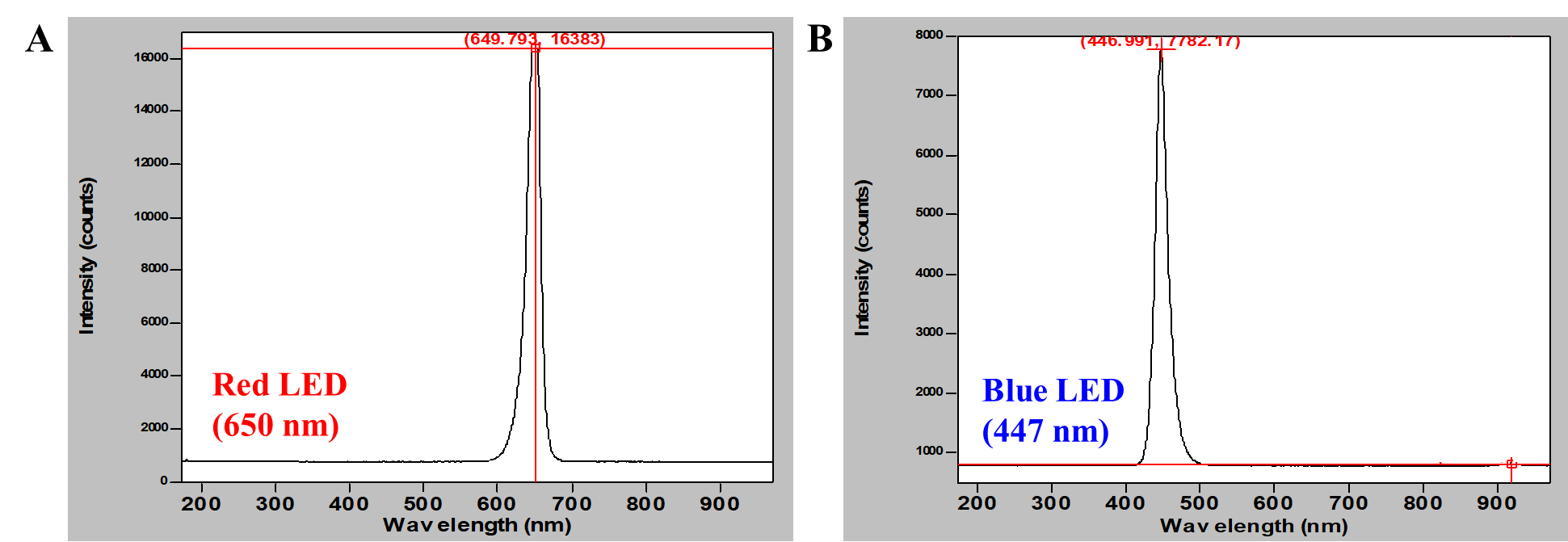


**Supplementary Figure 1.** Light spectra of the red (A) and blue (B) LEDs used in this experiment.


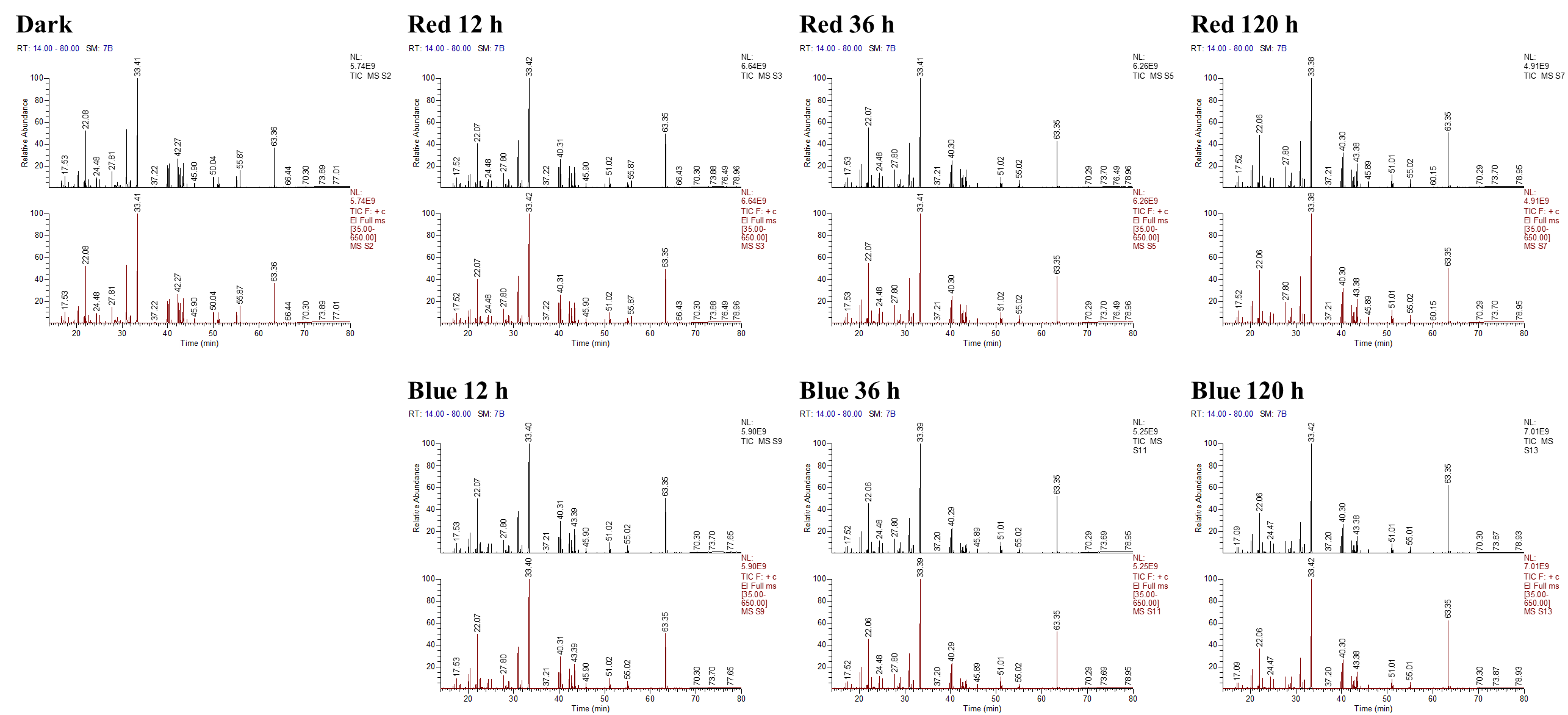


**Supplementary Figure 2.** Total ion chromatograms by GC-MS of soybean seedling grown under different light treatments.
